# Phylogenetic analysis and characterization of Korean orf virus from dairy goats: case report

**DOI:** 10.1186/1743-422X-6-167

**Published:** 2009-10-16

**Authors:** Jae-Ku Oem, In-Soon Roh, Kyung-Hyun Lee, Kyoung-Ki Lee, Hye-Ryoung Kim, Young-Hwa Jean, O-Soo Lee

**Affiliations:** 1 Animal Disease Diagnostic Center, National Veterinary Research and Quarantine Service, 480 Anyang-6-Dong, Anyang, 430-824, Republic of Korea

## Abstract

An outbreak of orf virus infection in dairy goats in Korea was investigated. Suspected samples of the skin and lip of affected goats were sent to the laboratory for more exact diagnosis. Orf virus was detected by electron microscopy and viral DNA was identified by PCR. To reveal the genetic characteristics of the Korean strain (ORF/09/Korea), the sequences of the major envelope protein (B2L) and orf virus interferon resistance (VIR) genes were determined and then compared with published reference sequences. Phylogenetic analysis revealed that the ORF/09/Korea strain was closest to the isolates (Taiping) from Taiwan. This is believed to be the first report on the molecular characterization of orf virus in Korea.

## Background

Contagious ecthyma (contagious pustular dermatitis; orf) is a common epitheliotrophic viral disease of sheep, goats, and wild ruminants and is characterized by the formation of papules, nodules, or vesicles that progress into thick crusts or heavy scabs on the lips, gingiva, and tongue. Orf virus is an oval, enveloped virus containing dsDNA genome within the genus *Parapoxvirus*, family *Poxviridae *[1]. The genus also includes pseudocowpox virus (PCPV) and bovine papular stomatitis virus (BPSV) in cattle and parapoxvirus of red deer in New Zealand. Zoonotic infection with orf virus is characterized by nodular and papillomatous lesions mainly on the hands, face, and mouth [2,3].

To reveal the genetic variation and characterization of parapoxvirus, the major virus envelope protein B2L and virus interferon resistance (VIR) genes have been used recently [4-8]. Orf virus infection has been diagnosed occasionally in Korea since the outbreak of orf was reported clinically in the 1990s. Although a few studies have been conducted, molecular epidemiology based on gene sequences of orf virus has not been performed because the population of sheep and goats is low in Korea. In the present study, orf virus infection in dairy goats was identified by clinical diagnosis and PCR. The complete B2L and VIR genes were sequenced, and their phylogenetic trees were constructed.

## Case presentation

**Figure 1:**
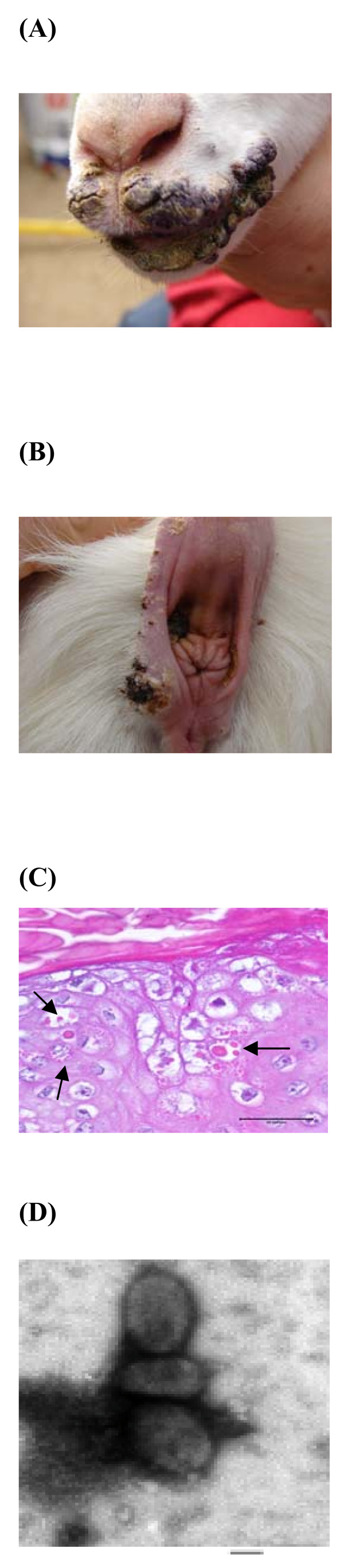
**Typical clinical cases of orf virus infection in diary goats (A, B), histopathological findings (C) showing intracytoplasmic eosinophilic inclusion bodies (arrows) in the keratinocytes indicated virus-induced lesions (HE, bar = 35 μm) and electron micrograph of the orf virus particle (D) showing typical oval-shaped morphology (bar = 100 nm)**.

In April 2009, an exanthematic outbreak occurred in a farm with 400 dairy goats in the Chungbuk province. Sixty dairy goats presented with wart-like lesions on the lips, tongue, and around the anus (Fig. 1A and 1B). The clinical diagnosis was contagious ecthyma. Morbidity was 15% (60/400), and goats of all the ages ranging from 2 weeks to 1 month were affected. Dried scabs collected from affected goats were stored in a -70°C freezer until the samples were used for further study.

The tissue samples were either fixed in 10% buffered formalin for histological examination, or were homogenized mechanically in PBS in a tube using a pellet pestle device. The homogenates were centrifuged at 3000 ***g ***for 5 min, and the supernatant was collected and negatively stained with 2% phosphotungstic acid for electron-microscopic examination of orf virus.

A PCR with OVS and OVA primers was performed as described previously [9]. In brief, the sequences of OVS and OVA primers are 5'-AGGCGGTGGAATGGAAAGA-3' and 5'-CCAGCAGGTATGCCAGGATG-3', respectively. The total viral DNA was extracted from scab samples using QIAamp DNA Mini Kit (Qiagen, Hilden, Germany) following the manufacturer's instructions. The amplication were performed in a GeneAmp PCR System 2700 (Applied Biosystems) using the following conditions: a preliminary heating for 5 min at 94°C; 10 cycles of 30 s at 94°C, 45 s at 55°C, and 1 min at 72°C; 20cycles of 30 s at 94°C, 45 s at 57°C, and 1 min at 72°C; and the final elongation of 5 min at 72°C. For phylogenetic analysis, a PCR was also performed using previously described primers [4,5]. The amplified gene was purified using an agarose gel DNA extraction kit (INtRON, Seongnam, Korea) and further cloned into pGEM-T vector (Promega, Madison, WI, USA) according to the manufacturer's instructions. Automated nucleotide sequencing of the VP60 gene insert was performed using an ABI 3130XL genetic analyzer with the BigDye^® ^Terminator cycle sequencing kit (Applied Biosystems, Foster City, CA, USA). The nucleotides at all positions were confirmed by three or more independent sequencing reactions in both directions. The published sequences of parapoxviruses were retrieved from GenBank for phylogenetic analyses (Table 1).

**Table 1:** Korean orf virus sequence and published parapoxvirus sequences used in the phylogenetic analysis

**Published Sequences**					
**Description**	**Country of origin**	**Species of origin**	**Reference**	**Region of gene**	**Accession Number**

ORF/2009-B2L	Korea	Goat	NA^a^	B2L	GQ328006
ORF/2009-VIR	Korea	Goat	NA	VIR	GQ328007
Orf-sh	USA	Sheep	Guo et al. (2004)	B2L	AY424970
Orf-ta	USA	Takin	Guo et al. (2004)	B2L	AY424971
Orf-mu	USA	Musk ox	Guo et al. (2004)	B2L	AY424969
Orf-va1	USA	Goat	Guo et al. (2003)	B2L	AY278209
Orf-ca1	USA	Goat	Guo et al. (2003)	B2L	AY278208
Orf-ca2	USA	Sheep	Guo et al. (2003)	B2L, VIR	DQ184476
NZ-2-1	New Zealand	Sheep	[13]Sullivan et al. (1994)	B2L	U06671
OV-IA82	USA	Sheep	[14]Delhon et al. (2004)	B2L	AY386263
Taiping	Taiwan	Goat	Chan et al. (2009)	B2L	EU327506
OV-SA00	USA	Sheep	Delhon et al. (2004)	B2L, VIR	AY386264
India 59/05	India	Goat	Hosamani et al. (2006)	B2L	DQ263304
Nantou	Taiwan	Goat	Chan et al. (2007)	B2L	DQ904351
Hoping	Taiwan	Goat	NA	B2L	EU935106
India 67/04	India	Sheep	Hosamani et al. (2006)	B2L	DQ263305
India 79/04	India	Sheep	Hosamani et al. (2006)	B2L	DQ263306
PCPV-TQ	NA	Cow	Guo et al. (2004)	B2L	AY424972
BPSV-RS	NA	Calf	Guo et al. (2004)	B2L	AY424973
BPSV-BV	USA	Calf	Delhon et al. (2004)	B2L, VIR	AY386265
1010/03 GR	Greece	Sheep	Kottaridi et al. (2006)	VIR	DQ275162
30/96GR	Greece	Goat	Kottaridi et al. (2006)	VIR	DQ275161
176/95GR	Greece	Sheep	Kottaridi et al. (2006)	VIR	DQ275165
928/02GR	Greece	Sheep	Kottaridi et al. (2006)	VIR	DQ275168
661/95IT	Italy	Sheep	Kottaridi et al. (2006)	VIR	DQ275169
12129/00IT	Italy	Sheep	Kottaridi et al (2006)	VIR	DQ275170
6126/02IT	Italy	Sheep	Kottaridi et al. (2006)	VIR	DQ275173
Orf-va2	USA	NA	Guo et al. (2004)	VIR	AY278210
1710/03GR	Greece	Goat	Kottaridi et al. (2006)	VIR	DQ275166
Orf-11-1	New Zealand	Sheep	McInnes et al. (1998)	VIR	AJ222702
Orf-11-2	New Zealand	Sheep	NA	VIR	AY292459
NZ-2-2	New Zealand	Sheep	[15]Mercer et al. (2006)	VIR	DQ184476
NZ-2-2	New Zealand	Sheep	[16]McInnes et al. (1998)	VIR	AF053969
MRI-Scab	New Zealand	Sheep	McInnes et al. (1998)	VIR	AJ222701
Orf-ta	USA	Takin	Guo et al. (2004)	VIR	AY424976
Orf-mu	USA	Ox musk	Guo et al. (2004)	VIR	AY424974
Orf-sh	USA	Sheep	Guo et al. (2004)	VIR	AY424975
155/95GR	Greece	Sheep	Kottaridi et al. (2006)	VIR	DQ275163
759/01GR	Greece	Goat	Kottaridi et al. (2006)	VIR	DQ275164
7389/03IT	Italy	Sheep	Kottaridi et al. (2006)	VIR	DQ275172
13598/03IT	Italy	Sheep	Kottaridi et al. (2006)	VIR	DQ275171
513/03GR	Greece	Goat	Kottaridi et al. (2006)	VIR	DQ275167
Hoping	Taiwan	Goat	NA	VIR	EU935104
Natou	Taiwan	Goat	[17]Chan et al. (2007)	VIR	EU327507
Taiping	Taiwan	Goat	Chan et al. (2007)	VIR	EU327508
GPDV	Taiwan	NA	Guo et al. (2003)	VIR	AF380126

^a^NA indicates not available

The B2L and VIR gene sequences of the orf virus, Korean strain were aligned with those of parapoxvirus sequences obtained from GenBank using Bioedit software (Ibis Biosciences, Carlsbad, CA, USA). A phylogenetic analysis was conducted using the Bioedit software and Molecular Evolutionary Genetics Analysis (MEGA) 3.1 software, with bootstrap values calculated from 1000 replicates [10]. The phylogenetic algorithm used for the building of the tree was the neighbor-joining method.

All clinical samples collected from the orf outbreak were positive using the previously described PCR method. The amplified PCR products were separated by electrophoresis and visualized by staining with ethidium bromide (EtBr). The size of amplified PCR product was 708 bp (data not shown). Keratinocytes in the stratum spinosum showed vacuolation and ballooning degeneration. Intracytoplasmic eosinophilic inclusion bodies were found characteristically in keratinocytes (Fig 1C). Negatively stained orf virus particles were examined by electron microscopy, which revealed the oval-shaped morphology of *Parapoxvirus*. The size of the observed virus particles was approximately 150-200 nm (Fig. 1D).

**Figure 2:**
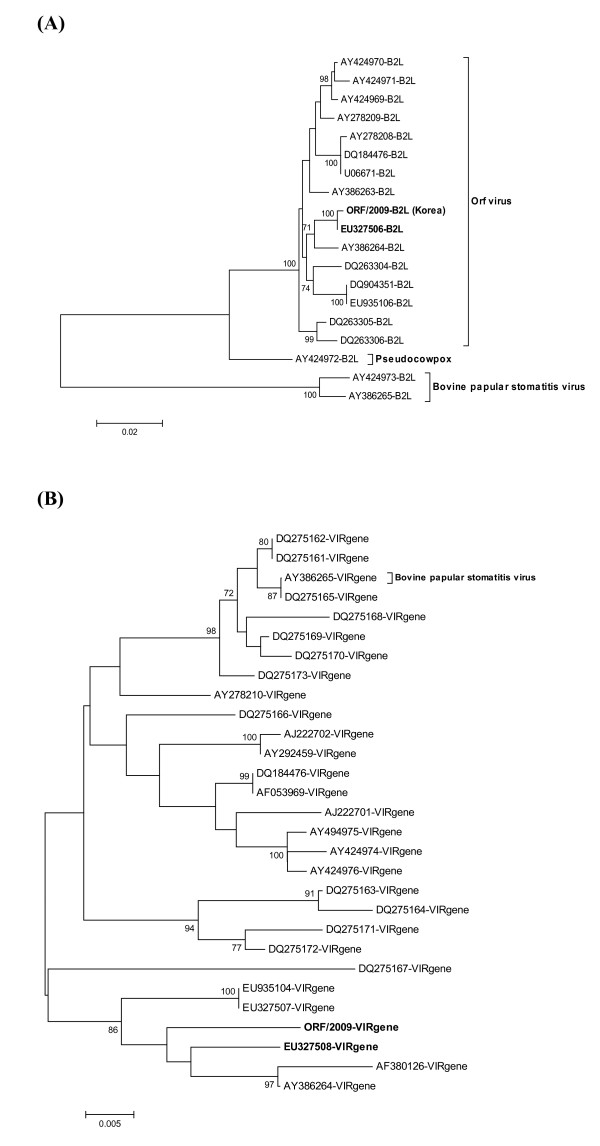
**Phylogenetic analysis of different parapoxviruses based on nucleotide sequences of B2L gene (A) and VIR gene (B)**. The nucleotide sequences of diverse orf viruses were aligned using the Bioedit program and Mega 3 software. One thousand bootstrap replicates were subjected to nucleotide sequence distance and neighbor-joining methods, and the consensus phylogenetic tree is shown. All bootstrap values are displayed above the tree branches, and only bootstrap values >70% are shown.

Additional PCR reactions were performed to obtain the full-length B2L and partial VIR genes. The amplified PCR products were 1206 bp for the full-length B2L gene and 552 bp for the partial VIR gene. The purified DNA was sequenced for comparative analysis. The sequence comparison of the ORF/09/Korea strain demonstrated a high degree of identity among our Korean strain and other orf virus strains. In phylogenetic analysis based on the complete B2L gene, the ORF/09/Korea strain was closer to the Taiping isolate (EU327506) from Taiwan (Fig. 2A). The ORF/09/Korea strain and Taiping isolate showed 99.82% and 99.48% similarity at the nucleotide and amino acid levels, respectively. The deduced amino acid sequences of the complete B2L gene of the ORF/09/Korea strain and the other strains were aligned using Bioedit software. Unique amino acid substitutions were found at two positions (A/R^250^→ D, I^275^→ M).

In phylogenetic analysis based on the partial VIR gene, the ORF/09/Korea strain also was closer to the Taiping isolate (EU327508) from Taiwan (Fig. 2B). The ORF/09/Korea strain and Taiping isolate showed 97.8% and 97.6% similarity at the nucleotide and amino acid level, respectively. The deduced amino acid sequences of the partial VIR gene of the ORF/09/Korea strain and the other strains were aligned, and unique amino acid substitutions were found at two positions (N/D^250^→ E, F^275^→ S).

## Conclusion

Orf infection is endemic in Korea, and no vaccination has been implemented to control this disease. An increase in the dairy goat population in Korea has been observed over the past several years. Clinical diagnosis and electron microscopy have been used routinely to identify orf viruses in Korea. Recently, PCR methods have been used to detect the viruses [11,12]. The dairy goat is not a native species in Korea and has been imported from abroad. Several causes of orf outbreaks exist, and the phylogenetic analysis may indicate a hypothetical origin of the viral strains involved.

Although only one case from dairy goats that showed clinical signs was used in the present study, the ORF/09/Korea strain was closest to the isolates (Taiping) from Taiwan, according to phylogenetic analysis based on the B2L and VIR genes. However, it is difficult to conclude the precise route by which the Korean orf virus strain was introduced into Korea based on molecular analysis alone. Also, at present, all ruminants from Taiwan have been prohibited from importation into Korea because of the outbreak of foot-and-mouth disease in Taiwan. Therefore, more epidemiological data concerning the distribution of orf viruses in Korea and neighboring countries are needed. Our continuing work to characterize more isolates in Korea and neighboring countries should prove valuable in that regard.

## Competing interests

The authors declare that they have no competing interests.

## Authors' contributions

JKO*, YHJ, and OSL participated in the planning of the project. KKL and HRK performed the PCR and phylogenetic analysis. ISR and KHL collected the samples and performed electron microscopy and histopathological examination. All authors read and approved the final manuscript.
